# Opportunities and Challenges for Improving Anti-Microbial Stewardship in Low- and Middle-Income Countries; Lessons Learnt from the Maternal Sepsis Intervention in Western Uganda

**DOI:** 10.3390/antibiotics9060315

**Published:** 2020-06-09

**Authors:** Louise Ackers, Gavin Ackers-Johnson, Maaike Seekles, Joe Odur, Samuel Opio

**Affiliations:** 1 School of Health and Society, University of Salford, Salford M66PU, UK; g.ackersjohnson@edu.salford.uk (G.A.-J.); m.l.seekles1@salford.ac.uk (M.S.); 2 Knowledge For Change, Bradford BD232HX, UK; joeodur@gmail.com; 3 Pharmaceutical Society of Uganda, Kampala 920102, Uganda; opixam25@gmail.com

**Keywords:** antimicrobial stewardship, pharmacy, sepsis, wound management, culture and sensitivity testing, resistance patterns, low-and middle-income countries, Uganda

## Abstract

This paper presents findings from an action-research intervention designed to identify ways of improving antimicrobial stewardship in a Ugandan Regional Referral Hospital. Building on an existing health partnership and extensive action-research on maternal health, it focused on maternal sepsis. Sepsis is one of the main causes of maternal mortality in Uganda and surgical site infection, a major contributing factor. Post-natal wards also consume the largest volume of antibiotics. The findings from the Maternal Sepsis Intervention demonstrate the potential for remarkable changes in health worker behaviour through multi-disciplinary engagement. Nurses and midwives create the connective tissue linking pharmacy, laboratory scientists and junior doctors to support an evidence-based response to prescribing. These multi-disciplinary ‘huddles’ form a necessary, but insufficient, grounding for active clinical pharmacy. The impact on antimicrobial stewardship and maternal mortality and morbidity is ultimately limited by very poor and inconsistent access to antibiotics and supplies. Insufficient and predictable stock-outs undermine behaviour change frustrating health workers’ ability to exercise their knowledge and skill for the benefit of their patients. This escalates healthcare costs and contributes to anti-microbial resistance.

## 1. Introduction

A recent review of research on antibiotic stewardship [1] found limited evidence of effective and feasible stewardship interventions in low- and middle-income countries (LMICs) and, where examples of effective interventions were identified, emphasised the essential need for contextualised. This paper reports on a recent, highly contextualized, facility-level intervention in a Regional Referral Hospital (RRH) in Uganda, known as the Maternal Sepsis Intervention (MSI). Funding for this action-research intervention came from the Commonwealth Partnerships for Antimicrobial Stewardship (CwPAMS) [2]. The funding body stipulated a focus on antimicrobial use (stewardship) and a project completion within 15 months with a budget of £60,000. The intervention was necessarily aligned with the Ugandan National Action Plan on Anti-Microbial Resistance or ‘NAP’ [3]. The NAP was launched in 2018 in an attempt to ‘slow down and contain’ [3] (p. 3) anti-microbial resistance (AMR). It sets out five Strategic Objectives focused on Awareness-Raising; Infection Prevention; Optimal Access and Use of Antimicrobials; Surveillance and Research. 

The CwPAMS objectives resonate most directly with Strategic Objective 3 of the NAP with a primary emphasis on Antimicrobial Stewardship (AMS). The NAP describes the use of antimicrobial agents as ‘the major modifiable driver of AMR’. According to the Plan, achieving optimal antimicrobial use ‘will require strengthening technical and regulatory frameworks, ensuring availability of appropriate medicines and changing behaviour amongst prescribers, dispensers and consumers’ [3] (p. 14).

This articulation of the funding body’s objectives with the NAP on AMR framed the design of the MSI. Building on strong pre-existing relationships especially in the field of maternal and new-born health, the project partners decided to focus the intervention on the Post-Natal and Gynaecology (PNG) ward in a RRH in Western Uganda. Fort Portal Regional Referral Hospital (FPRRH) has the second highest maternal mortality rate in the country. The most recent Ministry of Health report indicates a maternal mortality rate of 632/100,000 almost double the reported national average [4]. Sepsis competes with haemorrhage as the leading causes of maternal mortality [4,5,6] and Reinhart et al. [7] describe sepsis as a ‘Global Health Priority’.

Surgical site infection (SSI) following caesarean section contributes significantly to maternal mortality and morbidity [8] and to antimicrobial consumption. As a component of hospital acquired infection, it is also largely preventable. The decision to focus on the PNG ward reflected the opportunity to assess the potential for preventive intervention through improved infection prevention control (IPC) to reduce post-caesarean section SSIs. This focus also enabled us to address stewardship practices on a ward associated with the highest levels of antibiotic consumption in the hospital. 

The intervention built on the long-established Kabarole Health Partnership which involves a UK and Ugandan registered NGO (Knowledge For Change (K4C)) as the key operational partner together with the University of Salford; Kabarole Health District, FPRRH and the Pharmaceutical society of Uganda. The Health Partnership model has been actively developed through the Tropical Health and Education Trust (THET) as a more democratic and grounded approach to foreign engagement in global health. 

Substantial pre-existing research conducted in partnership with Knowledge For Change (K4C) has established the principle of co-presence to the achievement of effective knowledge mobilisation and behaviour change in health partnerships [9]. In practice, the mechanism involves the deployment of UK professionals working alongside Ugandan staff employed by K4C and local health workers in what can best be described as ‘knowledge mobilisation clusters.’ Long term continuity of engagement is the hallmark of K4C’s approach. Understanding the contextual dynamics of AMS is critical to behaviour change at individual and organisational levels. Capturing the effectiveness of this approach – based on continuous and active co-working—requires a longitudinal ethnographic methodology with in-built reflexivity. 

The MSI is reported in full in Ackers et al. [10]. This paper focuses on the mechanism that has supported the emergence of clinical pharmacy at FPRRH, and could form the basis of highly effective AMS. The development of this ‘mechanism’ has taken place over the past year. It has evolved in an iterative fashion as part of a continuous, exploratory, journey supported by on-going ethnographic co-researching. This type of approach does not lend itself to a linear, before-and-after, hypothesis testing structure. The paper instead charts the evolution of the MSI and the data collected along the way. As data presents new theories, this then creates new opportunities for data collection. 

The intervention team started with a focus on SSI wounds, which led to an initial emphasis on wound care. Nurses and midwives are the custodians of wounds in Ugandan public facilities. The quality of wound care was found to be grossly inadequate at project inception; wound dressing was infrequent, and health workers were avoiding this task. Improvements in wound care led by nurses and midwives created the opportunity for swabbing and laboratory testing. Active engagement between nurses, midwives and laboratory scientists then created the evidence base, stimulating the opportunity for highly effective and impactful clinical pharmacy and multi-disciplinary team working. The first part of the paper tracks this process. Ultimately the effectiveness of this team in achieving optimal AMS is limited by access to antibiotics and IPC supplies. The second part of the paper presents data evidencing the dynamics of supply chain failures in the Ugandan public health system.

## 2. Methods

The approach can best be described as a multi-method ethnography, commencing with observational work on the ground. Observational work was undertaken on a co-researching basis with a lead role played by Ugandan staff employed through K4C, supported by repeated and extended site visits by the Principal Investigator and virtual co-presence over a 15-month period. The team were joined by the Ugandan lead and attended Hospital IPC meetings on two occasions. Observations, complemented by on-going WhatsApp and Skype conversations were recorded in notebooks, minutes, reports and emails, and entered into NVIVO for storage and analysis.

This observational research generated theory inductively which, in turn, stimulated the search for other sources of data. Although we had anticipated accessing facility data on antibiotic consumption we could not have known or understood the complexity of this process and the challenges of even defining consumption in a public hospital setting prior to the start of the project. In such situations and given the essentially inductive quality of ethnographic research, where context is ‘everything’ [11], a simple a priori (deductive) hypothesis setting is inappropriate. In that respect, a process of conceptualisation, theory generation and data collection took place simultaneously. Every attempt to record or collate data stimulated intense on-going discussions about the recording processes and the nuances of its interpretation. In most cases it led us to new lines of enquiry (theories) and approaches to data collection. Much of the data, as is normal in this context, was not collated and had to be manually and painstakingly searched for from casefiles or record books. The very poor quality of documentation in patient files and subsequent records management is a critical dimension of context with implications for AMR [8]. Data collection became a process of exploration, involving forms of local capacity-building along the way on methods of organising and storing hospital records and entering them into excel spreadsheets. In this context (as in many others), much of the facility-based data could not be interpreted at face value as facts; but rather, as artefacts reflecting their (social) construction. 

Facility data has been collected from a wide range of sources. Firstly, data on drug orders and supplies from National Medical Stores (NMS), was obtained through an on-line national pharmacy data base, known as the Rx system, the use of which was functionalised through the project. This was supplemented by data from paper-based records (the Dispensing Log) of supplies distributed from the central hospital stores to the wards over a 4-month period, from December 2019 to April 2020. Further, the Infectious Diseases Institute (IDI) supported hospital laboratory have proved key partners both in the intervention itself, with laboratory results providing a critical stimulus to multi-disciplinary team working, but also in generating research data. This commenced prior to the project as part of Ackers-Johnson’s microbiology doctorate [12] and has continued throughout, generating valuable data on resistance patterns. The laboratory provided data on test results of samples taken from the PNG ward in 2019. This complemented a data set generated from 142 cases of suspected sepsis between January 2019 and February 2020 that were identified through a manual search of paper-based patient records.

In January 2020, a phase of qualitative interviewing took place to capture perceptions of the impact and effectiveness of the intervention. Twenty-five interviews were conducted with all cadres involved in the MSI, including 50% of the nurses, midwives, intern doctors, laboratory technicians and pharmacists working on the PNG ward, two hospital managers and three UK volunteers. The interviews were transcribed and thematically analysed using NVivo 12. Ethical approval for the work was gained from the University of Salford, Makerere University and the Ugandan National Council of science and technology (HS249ES).

## 3. Results and Discussion

The MSI built on on-going under-pinning research including three PhD studies. One of these (Ackers-Johnson) involved active co-researching on emerging AMR patterns with microbiologists in the hospital laboratory. The team was aware that the laboratory was struggling to obtain adequate samples from the hospital wards for testing and that maternal sepsis was one of several priorities for their research.

In common with all projects funded by THET, the CwPAMS funding stream identified a knowledge transfer mechanism based on the harnessing of UK (National Health Service) health worker expertise as the basis for behaviour change interventions. The role and contribution of professional volunteer engagement has been researched extensively by the authors, with an emphasis both on the impacts on LMICs [9,13] and, in a study financed by Health Education England, the benefits to the NHS [13]. Our approach to the MSI was informed by this research and resulted in the decision to deploy professional volunteers in co-working, mentoring roles for the duration of the intervention. The aim was to have continual presence on the ground with UK volunteers working alongside locally recruited staff (through K4C) and health workers in the hospital. One of the volunteers recruited was a member of the Ugandan diaspora working in the NHS. This volunteer knew the region, spoke the local language and specialised in wound care and SSIs. The importance of creating the conditions for serendipitous opportunities to influence action-research interventions has been reported elsewhere [14,15,16]. 

### 3.1. The Maternal Sepsis Intervention and Wound Management as the Focus for Change

The MSI proposal made no specific reference to wound care; wounds were something to be swabbed in order to test resistance patterns, and we had not anticipated the value of wound care to AMR work and patient outcomes. The early decision to focus on post c-section wounds turned out to be pivotal; it encouraged a very grounded approach focusing on multi-disciplinary team working at the patient’s bedside. The lack of effective wound care was found to be contributing to extended patient stays and inappropriate use of antibiotics. More immediately, the ward had become associated with the stench of infection; nurses and midwives were reluctant to spend time with patients with badly infected wounds. They considered the work to be unpleasant and, in the absence of hand washing and protective clothing, staff feared the risk to their own health. The initial engagement in wound cleaning and dressing by the K4C midwives, the UK volunteers and a pioneering local midwife stimulated an holistic investment in IPC measures. This very quickly delivered major and very tangible results: Critically, it created a safer and more comfortable environment for wound swabbing. Working closely with our hospital laboratory colleagues, the project began to see a transformation of practice from a situation where no wounds were being swabbed (or other samples taken) to one where wounds were being dressed (and seen) twice daily; all patients with suspected infections were being identified, having samples taken and sent to the laboratory for culture and sensitivity testing. Table 1 presents the results of data collected from case files of all suspected sepsis cases in the 12 months commencing 1st January 2019. They show the lack of swabbing and culture and sensitivity testing on the wards prior to project commencement. The implementation aspect of the project began to impact in July, after a short initial transition period with few cases swabbed. After 22nd July 2019, nearly all suspected sepsis cases have been identified and samples sent to the laboratory for testing.

Laboratory results from these tests were present in the files of 67 of the 74 (90.5%) patients who had had a swab taken. For four patients, the results had gone missing from the file; for two patients the test was not completed because the IDI hospital laboratory was closed over Christmas and New Year and one patient’s lab test was not completed because the patient had discharged herself against medical advice. Although this emphasises the importance of improving record-keeping, this level of documentation represents a remarkable achievement in the context.

The impact of the focus on wound care and culture and sensitivity testing is explained by a local midwife. She had taken a particular interest in the use of sugar in wound care prior to the project (in Sudan) and had previously worked alongside K4C staff and British nursing students on the labour ward, so relationships were strong. She describes the impact the project has had on her personally and on the ward and patients. She notes that, prior to the project, empirical prescribing of antibiotics lacked the desired effectiveness, and this lack of effectiveness was compounded by prolonged prescribing of the same antibiotics. Importantly, she also specifically recognises the role that clinical pharmacists are now playing:

You came in at a critical time [and] brought new skills. Before there was no culture and sensitivity testing. Some of us knew about it but had never used it—even the doctors. When you came in it is me who benefitted most; I was carrying a very heavy burden and you helped me. You came as a combined team. We have not lost any women from sepsis since the project started and Dorothy (a Ugandan midwife employed by K4C) came. I had worked with her on labour ward with your students. Even the laboratory has started to respond—the burden was lifted, and everyone started getting involved.

We did use culture and sensitivity tests in Mulago (National Referral Hospital) but with not much emphasis and sometimes you have your interests on other things and we left it to the doctors. Here much of the things are now done by nurses/midwives—like doing culture and sensitivity tests. We knew culture and sensitivity would get results. Now I try to do the septic patients first. Before we noticed some were not getting better and we did not pay much attention to how this woman has been on this treatment for so long and you just gave her more antibiotics. […] now [the pharmacist] comes on the ward daily and looks around and helps us as sometimes the intern doctors are busy and lack supervision. Before we used the same medicines—same—same—we just gave what was prescribed.

In addition to describing the importance that swabbing and testing has made to progress, the midwife alludes to a major change in team-working and task-shifting with midwives and nurses now playing a very central role in these processes. This has been critical to the effectiveness of the MSI. Midwives and nurses are barely mentioned in the NAP. In the Ugandan context their active engagement and empowerment is absolutely essential to AMS, not least because they are most often the only cadres continually present on the ground. The presence of senior doctors is at best sporadic with rotating and largely unsupervised intern doctors providing most medical input [10,17,18]. This evidence adds weight to Brink et al.’s proposal for new nurse-led models of AMS in Africa [19].

The following section examines how the presence of laboratory results has created the opportunity for improved antimicrobial stewardship through clinical pharmacy engagement.

### 3.2. AMS Performance Indicators: The Engagement of Clinical Pharmacy at FPRRH

One of the key AMS performance indicators identified by the pharmacy team at FPRRH is the ‘Review of Pharmacotherapy’ by pharmacists. In practice we are concerned here with the extent to which pharmacists are directly engaged in multi-disciplinary decision-making following the receipt of laboratory test results showing resistance patterns. The data collated from patient notes showed that pharmacists reviewed the pharmacotherapy in 91.8% of cases where test results showed a bacterial growth. 

We must not underestimate the impact of the introduction and embedding of culture and sensitivity testing to the team-building process. Test results trigger team-based activity; they engage all staff irrespective of cadres. Having the laboratory results provides a focus for interests to coalesce around; they stimulate team discussion and active pharmacy engagement and create the environment for genuinely patient-centred care. Having the evidence-base for rational prescribing undermines entrenched disciplinary hierarchies. It was clear from the discussion in a hospital-wide Infection Prevent Control (IPC) Committee that the tension between doctors and pharmacists persists in other areas of the hospital and had been evident, on occasion, on the PNG ward:

Sometimes you find a pharmacist has changed a prescription and then on the ward round the doctor changes it back to a drug the patient is resistant to. (Midwife)

A pharmacy intern responded to this comment:

I think it should be teamwork here and respect for each other. If we advise and then the prescription is changed the clinicians come and undermine that decision without listening to the pharmacist. We can see that post-natal is taking the lead in consulting with pharmacy, but other units are relying on empirical usage designed for health centres and not for hospitals. If you are rigid on the usage you will not use the pharmacists/laboratory’s advice.

Evidence of a transformational increase in direct clinical pharmacy engagement on the wards is supported by qualitative findings. One midwife refers to the impact of laboratory results on these hierarchies:

Sometimes there can be ego—that the doctor or pharmacist thinks, ‘I am the overall boss so I can’t be directed on what to do’, but with the data that goes down.

Every respondent identified the improvement in teamwork and identified this as the source of change on the wards:

We are now working hand-in-hand with the pharmacists, the laboratory and the doctors—in-charge nurse.

An intern doctor also describes how useful he has found the expertise of the pharmacy team:

It’s changed a lot now; the senior pharmacist comes regularly, and you may find there are 2 microorganisms sensitive to different antibiotics. Now I don’t have the time to walk to the pharmacy and those people have studied medicines. I have textbook knowledge; if someone has a UTI (Urinary tract infection) I give x. I did study this but as time goes on you get used to giving certain medicines quite often and you are not so equipped to understand how one medicine interacts with another one or if a patient has TB or is HIV positive or how to combine drugs—so a pharmacist being available on the ward has really brought in great improvement.

A midwife shows her appreciation of the teamworking environment that has developed. She refers to the presence of pharmacy on the ward and the lengths the pharmacists have gone to, to try to secure appropriate antibiotics:

It has greatly improved because right now we have pharmacists who come on a daily basis or if not, every day we can’t go 3 days without seeing one who can guide us on the mothers and which drugs to take. They interact with the doctors; if you don’t interact there is that collision. Right now, there is no tension. They say, ‘what if we do this’ and there is a discussion. We never used to have any pharmacists coming on the wards, so it was majorly the doctors dealing with the prescriptions. We have managed to reduce the irrational use of antibiotics.

The use of language in this response by the midwife illustrates the growing status of midwives and nurses in the multi-disciplinary teams and their ability to talk confidently about ‘rational’ prescribing; a concept they would not have been aware of prior to the MSI. It also illustrates the ‘boundary spanning’ role nurses and midwives are playing on the wards mediating professional hierarchies and tensions. Pharmacists were also very aware of their role in mediating these boundaries, and take care not to ‘clash’ with doctors:

[Pharmacy] don’t see all the cases. We try to pick cases where we feel pharmacists can have an input and that way, we don’t clash so much with the doctors. We work within our mandates so there are no clashes. Intern doctors are also using this as a chance to learn about AMR.

The MSI has achieved optimal pharmacy engagement (in an RRH context) on the PNG ward. The impact of laboratory testing has played an important role in empowering pharmacy. This is evident in the new policy, initiated by pharmacy with strong support from the laboratory, of only permitting use of high-end antibiotics when laboratory test results are available. A midwife respondent makes the point that there is a limited role for pharmacy on the ward in the absence of laboratory results:

Pharmacy will tell you there is no point in them coming unless there are cultures. Clinicians are not allowed to change antibiotics now without cultures.

Discussions have taken place in the hospital’s IPC committee to extend this policy to all wards, illustrating the wider impact of the MSI on the hospital as a whole. The laboratory respondent welcomes this achievement, which also represents the growing recognition of the pharmacy presence in the hospital:

The policy of only prescribing high end antibiotics to patients who have had culture and sensitivity testing has really worked; these antibiotics are being guarded jealously now. In fact, (the pharmacy team) are very strict on that. I really feel this could work on other wards. It is only working on post-natal ward at present because they have laboratory reports.

### 3.3. Creating the Evidence Base and Momentum for a Hospital Antibiogram

Another important aspect of this wider impact can be seen in the role that the MSI has played in creating the evidence base for a hospital antibiogram. An antibiogram is a collection of data, based on laboratory testing of the pathogens in a specific facility that summarises patterns of resistance to different antimicrobial agents (or antibiotics). Although international and national trends in resistance patterns can be identified, regional and facility-specific patterns enable even closer targeting of antibiotics. 

Whilst we have seen the benefits of culture and sensitivity testing in terms of trying to identify the optimal antibiotic for individual patients; in cases of suspected sepsis, health workers cannot wait for the test results, but must immediately start the patient on an antibiotic, whilst awaiting the testing process. This is known as ‘empirical prescribing’. In FPRRH (as in many other facilities), the prescribing decision, usually made by a junior doctor, will be based on their usual practice, perhaps with reference to the formulary—and is very much tempered by their perception of what is available in stores. Where a hospital antibiogram exists, this initial empirical prescribing can be informed by local evidence and has a much higher chance of success. The presence of an antibiogram with associated awareness raising and sensitisation amongst all staff, and especially medical interns, would have major impacts on empirical prescribing across the hospital. Prior to the MSI, FPRRH did not have the volume of laboratory results to create the necessary evidence base for a hospital antibiogram. A member of the pharmacy team describes how this has changed:

If you go to maternity, you will notice a very big change. The ward sends the biggest volume of swabs now to the laboratory because those people [midwives] are aware.

The laboratory scientist confirms this:

On the basis of the increased swabbing we hope to be in a position to have an antibiogram. This will be very informative—the sample size is now very adequate, but we want to enter this information into a comprehensive database which has different parameters—length of stay–age-sex-ward–so that when you are doing the analysis it is very comprehensive. The antibiogram will be good for the clinicians to guide prescribing—it will be good for the patients.

The results presented above demonstrate the ability to make considerable progress in AMS at a RRH. 

### 3.4. Access to Antibiotics in FPRRH: Supply Dynamics

Ultimately, the model that has evolved on PNG ward has the potential to significantly reduce infection, improve prescribing practice, reduce antibiotic consumption and overcome some of the effects of AMR on patient outcomes. The major challenge facing the MSI model is access to the right antibiotics and antimicrobials at the right time. The laboratory scientist is clear about this:

Antibiotic stock-outs remain a serious constraint; in many cases patients can only be given the right antibiotics if they pay and many of them can’t pay. We have to be very clear, antibiotic stock-outs are a key factor fueling AMR. If we look at the scenario where we have investigations done and antibiotics are available, and the outcomes are good, but we have done the investigations and the antibiotics are out of stock we won’t have a good outcome.

There is a bigger concern here too; if supplies are not available and the ward staff are unable to respond effectively to laboratory results this can be predicted to have a major impact on staff motivation and the behaviour change gains. Problems of access critically restrict pharmacy’s ability to engage in rational prescribing; prescribing the drug most likely to work according to laboratory results. This in turn leads to over-consumption of poorly performing antibiotics and poor patient outcomes. The following section describes the supply chain system at FPRRH and presents data on consumption. Critical problems include:

1. The hospital may only order against a budget prescribed by the Ministry of Finance and held by NMS.
2. The hospital can only order antibiotics from a prescribed catalogue which excludes many of the antibiotics indicated as necessary from laboratory results and present on the ‘Essential Medicines and Health Supplies List for Uganda’ [20].
3. There are major and unpredictable discrepancies in what is ordered and what is delivered (‘Order Fill Rates’).
4. As a result of the above, most IPC supplies and antibiotics run out half-way through the bi-monthly supply cycle (Stock-Outs).

### 3.5. The Impact of the MSI Project on the 2020/2021 Procurement Plan

In Uganda, the funds for procurement of drugs and supplies in the public sector are highly centralised and inadequate. NMS procures and distributes supplies to health facilities based on a centrally allocated Annual Supplies Budget. Each hospital is required to produce an Annual Procurement Plan. Once agreed, this Plan is fixed and cannot be varied over the year reducing the opportunity for flexibility and responsiveness to the hospital laboratory results and any changes indicated by a future antibiogram. This budget is held by NMS. With the exception of private wards, it is not possible for a RRH to source supplies from elsewhere. NMS deliveries take place bi-monthly.

During the annual procurement process, the hospital may only order those items authorised by the ‘Essential Medicines and Health Supplies List for Uganda’. However, not all essential drugs feature in the NMS catalogue. A hospital pharmacist describes the situation as follows:

As much as we may desire a certain antibiotic, we can’t plan for it if it is not present in the catalogue. A case in point is Amikacin and Moxifloxacin.

Out of the nine antibiotics tested against *Acinetobacter* samples in the laboratory, only two—doxycycline and amikacin—showed greater levels of susceptibility than resistance (for details of these test results, see Supplement 1). Given the much higher success rate of amikacin, it is paramount that the antibiotic can be obtained for cases of severe *Acinetobacter* infections where other avenues have failed.

**Figure 1:**
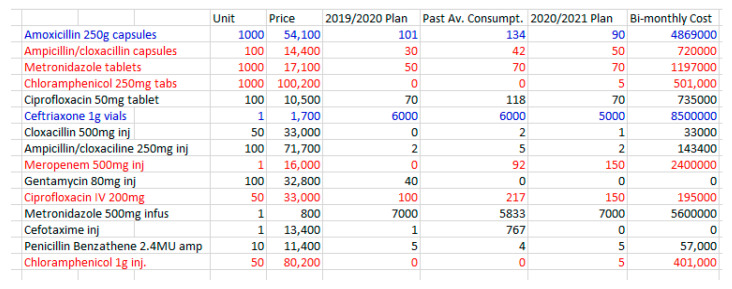
Extract from the 2020/2021 Procurement Plan (with bi-monthly figures) focusing on key Antibiotics used on PNG Wards. In this table, the column ‘unit’ shows the number of doses per unit as sold. ‘Price’ is the price per unit in Ugandan Shillings (1 USD = 3780 UGX, May 2020). The column ‘2019/2020 plan’ shows the number of units ordered for delivery every other month in 2019, with ‘past av consumption’ detailing the average bi-monthly consumption of the past year. The column ‘2020/2021’ details the set number of units ordered every other month for this year, with the corresponding bi-monthly cost reported in the final column.

Within the constraints described above, the MSI has influenced procurement planning for 2020/21. Figure 1 evidences significant changes in antibiotic ordering and consumption arising directly from the intervention, where antibiotics in red denote project-related increases and those in blue denote decreases.

The pharmacy team involved in procurement planning pointed out the severe budgetary constraints they faced when attempting to order new antibiotics in response to laboratory results. In practice, this meant making difficult ‘trade-offs’, especially when the new antibiotics are so much more expensive than those they were able to reduce. The reduction in supply of amoxicillin for example is explained as follows:

We realised that the majority of patients using amoxicillin were mothers discharged after giving birth. They are usually given amoxicillin as prophylaxis to prevent infection. Some were being given for a longer duration than necessary. As pharmacy staff, we intervened so that the duration of treatment would be reflective of the nature of risk. This led to a reduction in use. We had to increase certain antibiotics or include new antibiotics as well. Due to budget constraints, it was agreed during the planning stage that we cut on the quantity of Amoxicillin to free up some budget to cater for other needed antibiotics.

The laboratory results indicated very high levels of resistance to both amoxicillin and ampicillin, both of which are derived from penicillin (Supplement 1). Based on these assumptions, ampicillin and amoxicillin will have minimal effects on the three primary bacterial causes of infection.

Significant changes in planned use of Meropenem can also be directly attributed to the MSI. The pharmacist explains that consumption of this drug over the past year has been reliant entirely on donated supplies (it was not ordered in 2019):

[The increased order] can be supported by evidence generated by the laboratory. Due to the increased culture and sensitivity reporting, we noticed that there was improved sensitivity to meropenem. This ensured that we were able to convince members involved in planning to include it on the 2020/2021 plan. We were able to get some donations last year and that’s why it shows that we consumed it. What’s more, we wrote to NMS to allow us procure it, even though it’s not in the current plan.

This action, of communicating directly with NMS on procurement, represents one example of a scenario where the pharmacy team have attempted to advocate as a result of the MSI. The impact of this procurement may be to the benefit of other hospitals if NMS are influenced to place it on their catalogue in future.

The decision to increase orders of Meropenem required the team to make stark choices, which led to the reduction in orders of cefotaxime:

Just like Meropenem, this particular consignment of Cefotaxime (used in 2019) was a donation. It wasn’t in the procurement plan. While working on the 2020/2021 plan, we had to prioritise between Cefotaxime and Meropenem. We had to go with Meropenem. We did factor in the cost and resistance profile per the lab reports.

Cefotaxime has shown high levels of resistance in the laboratory tests (Supplement 1). The marked rise in procurement of Ciprofloxacin is also directly attributable to the MSI, although the pharmacy team were concerned about the volume needed:

What we require is actually a lot more. Again, [the increase] can be explained by the results of culture and sensitivity. There seems to be less resistance to ciprofloxacin.

The pharmacist sums up the impact that the project has had on procurement planning and the constraints the team had to work with:

We had to reduce the quantity of Ceftriaxone by a significant margin. This again was supported by laboratory data which showed a lot of resistance to ceftriaxone. Some of the monies freed up were used to plan for chloramphenicol and meropenem, drugs which are showing less resistance as per lab reports. There is no significant increase in this current budget and the incoming budget for drugs and medical sundries. It’s therefore painstaking to reallocate priorities in terms of drugs while maintaining the same budget. Our [MSI] efforts to encourage and support Culture and Sensitivity testing and sharing this with the procurement planning team lead us to include some much-needed antibiotics (Meropenem and Chloramphenicol) on next year’s plan and reduce the procurement of antibiotics with a lot of resistance (Ceftriaxone).

Figure 1 also provides an indication of the cost implications of the changes in antibiotic procurement as a result of the MSI. Most of the increases in procurement involve more expensive antibiotics. The procurement plan reflects the negotiations the pharmacy team have engaged in, to balance the need for rational prescribing against the cost implications of buying more expensive antibiotics. Unfortunately, the constraints of the NMS budget-line are not the end of the story.

### 3.6. Discrepancies between Order and Supply (Order Fill Rates)

In practice, not all that is ordered by the hospital from NMS is supplied. The ‘Order Fill Rate’ gauges the delivery performance of total number of items ordered against the total number of items delivered. As clearly seen in Table 2, NMS supplies about 75% of orders. More specific discrepancies may also arise. Unusually, NMS failed to deliver Ceftriaxone in September 2019, for example.

### 3.7. Key Challenges to Sustained Behaviour Change: The impact of Stock-Outs on AMS

Stock-outs (the exhaustion of supplies) are a feature of Ugandan public health facilities at all levels, and are a major factor contributing to sepsis deaths in maternal and new-born health [21]. Inevitably, the bi-monthly deliveries tend to be exhausted quite rapidly and often by the end of the first month, when ‘stock-outs’ become a major feature of life and cause of morbidity and mortality at FPRRH. A pharmacist describes the situation as follows:

For example, when we get 6000 vials of Ceftriaxone, we consume all of it in maybe 4 weeks, then we stay without for another 3–4 weeks. And the following cycle, we get the same quantity. Therefore [consumption data] are merely an average of what is not enough.

Stock-outs are caused by a combination of misuse and overall shortfalls. The pharmacists noted that the project had improved antibiotic use on the wards:

When these antibiotics are received [from NMS] they tend to run out quickly. Again, this is attributed to the small budget and probably misuse/irrational prescribing. However [MSI’s] endeavour to link the ward, pharmacy and the Lab has to a great extent solved the issue of irrational antibiotic prescribing.

By way of illustration this shortfall, on 18th February 2020 the PNG ward contacted K4C to request support in the purchase of gauze. Without this, they would not be able to continue with the wound dressing established on the ward. This would have resulted in increased infection and sepsis (and antibiotic consumption). We were aware during the project visit in January 2020 that the hospital had also run out of disinfectants, iodine and spinal needles (amongst many other things). In such circumstances the only option is for staff to ask patients to pay for the necessary items, and if they are unable to pay then operations will not happen, and major delays occur in treatment. On 19th February 2020 we established that 13 key items for use on the PNG ward were out of stock and had been for over a month:

1. Ceftriaxone injection;
2. Intravenous metronidazole;
3. Intravenous Normal Saline;
4. Intravenous Ringers Lactate;
5. Intravenous Ciprofloxacin;
6. Meropenem 1 g injection;
7. Gentamicin 80 mg injection;
8. JIK (Sodium hypochlorite) solution;
9. Alcohol hand rub;
10. Chlorhexidine Gluconate;
11. Cotton Wool 1 kg (hospital quality);
12. Gauze (Hospital quality);
13. Povidone Iodine;

The next supplies were expected on 25th February.

### 3.8. Antibiotic Consumption Patterns at Ward Level in FPRRH

**Figure 2:**
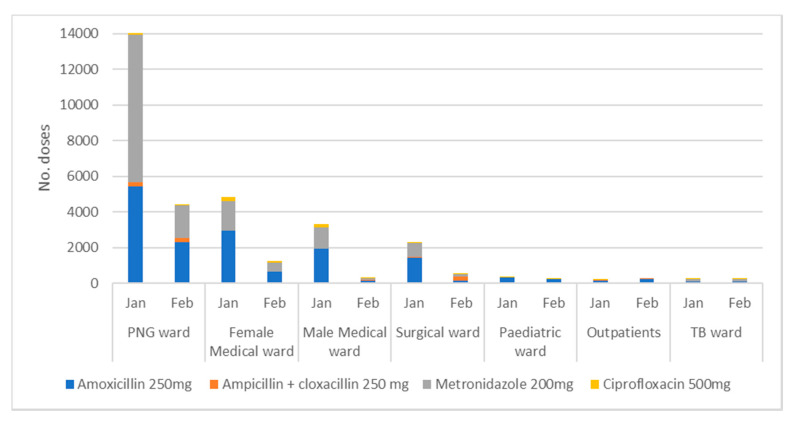
Supply of Oral Antibiotics to All Wards in January and February 2020.

When supplies arrive at FPRRH from NMS, they are located at the Main Stores. At this point, supply data is recorded electronically in the on-line ordering system (Rx). The process of distributing supplies within the hospital is, unfortunately, not covered by this electronic system. Instead, the in-patient pharmacy (located a short distance from the Stores) orders from the Stores. Individual wards then visit the in-patient pharmacy to requisition supplies, and this is recorded manually on forms and in a records ledger book (the HMIS Dispensing Log). Figure 2 presents data on the distribution of oral antibiotics between the main hospital wards in January and February 2020. As can be seen, the level of antibiotic consumption on the PNG wards as a proportion of overall consumption is high and indicates the importance of this to overall AMS. The data presented in this figure illustrates three important trends, which were also seen in the use of intravenous antibiotics:

- The dominance of Amoxicillin and Metronidazole in antibiotic consumption.
- The significant contribution that PNG ward makes to overall antibiotic consumption.
- The profound impact of stock-outs on access to antibiotics with a reduction in oral antibiotic use in February, showing a reduction in consumption to between 25% and 30% compared to the January figures.

**Figure 3:**
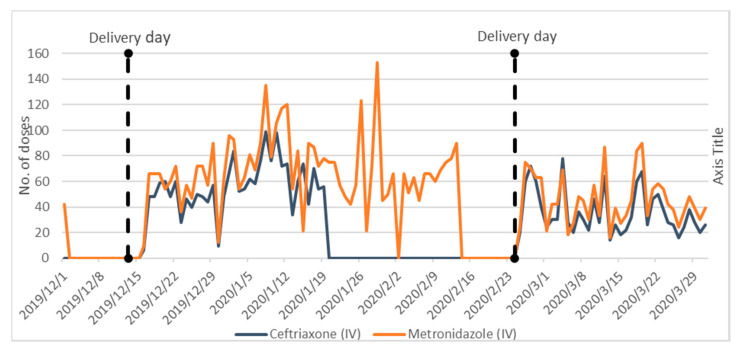
Dispensing of IV Ceftriaxone and Metronidazole from In-Patient Pharmacy to Post-Natal and Gynaecology (PNG) Wards (1/12/2019–29/03/2020).

**Figure 4:**
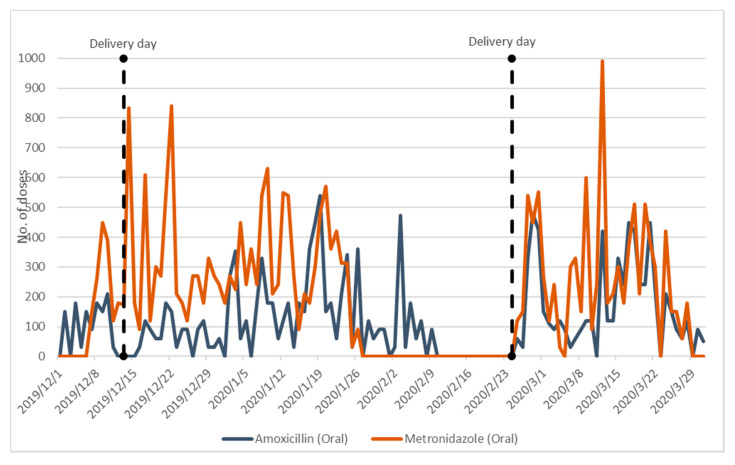
Dispensing of Oral Amoxicillin and Metronidazole from In-Patient Pharmacy to PNG Wards (1/12/2019–29/03/2020).

Figure 3 and Figure 4 present data collected from the in-patient pharmacy on key antibiotics used in the PNG wards only. NMS deliveries were made on 13th December 2019 and 24th February 2020. The dispensing patterns show stark evidence of stock-outs. 

The higher consumption of metronidazole can be attributed to the high dosing regimen (three times daily), plus its empirical indication as a broad-spectrum therapy for prophylaxis against anaerobes. IV Ceftriaxone (dosed once daily) is also being used empirically and for prophylaxis, especially in surgical cases. Dispensing of IV metronidazole showed evidence of stock-outs but for shorter periods than Ceftriaxone. In the period between 15th and 23rd February, neither IV Metronidazole or IV Ceftriaxone was available to the PNG wards. Similar falls can be seen at Christmas and New Year.

Dispensing patterns for oral metronidazole show higher utilisation and longer periods of stock-outs than oral amoxicillin, with an extended stock-out from 26th January to 23rd February coinciding exactly with the stock out of IV Metronidazole.

### 3.9. Interpreting In-Patient Pharmacy Data—A Note

We discussed some of the challenges of collating and interpreting facility consumption data (above). The emphasis in instruments such as the WHO’s Practical Toolkit for AMS programmes in health-care facilities in low- and middle-income countries [22], which prescribe ‘outcome measures’ aligned to Western consumption indicators (such as Defined Daily Dose and Direct Observed Therapy) fail to capture the reality of many LMIC contexts. Hantrais’ work on comparative methods [23] discusses the idea of ‘conceptual equivalence’, arguing that ‘concepts cannot be separated from contexts’ [23] (p. 73). Operationalising the concept of direct observed therapy presents insurmountable challenges in Ugandan RRHs. Hospital pharmacists were very aware of these limitations:

We cannot measure performance by zeroing on antibiotic [consumption] only.

Collecting and analysing the data on antibiotic ‘consumption’ patterns at FPPRH has emphasised the dangers of empiricist approaches to data analysis and presentation, and the importance of interrogating data rigorously. In most cases, data presents a myriad of questions and very few obvious and immediate answers. This is especially the case when attempting to collate data from public health facilities in Uganda and many other LMICs. Collecting data has been a continuous process of trial and error, merging with the underlying ethnographic journey. As described, in the case of in-patient pharmacy, the data is not yet managed electronically via the Rx system. Rather, individual wards come to a window in the in-patient pharmacy with paper forms requesting supplies for that day. The pharmacy then maintains a hand-written record of dispensing in a records book and this data is compiled into forms for the Ministry of Health. In the first instance, we believed that all wards behaved in this manner, but the initial data suggested otherwise. The TB ward, for example, appeared to collect very few drugs. We found that most of the drugs used on the TB ward are in fact provided by a donor and follow a different track. These drugs pass through the main stores and are directly requisitioned by the TB ward. As such, they do not pass through in-patient pharmacy and neither are they recorded as received from NMS on the Rx system, leaving a gap in overall supply and consumption data.

Our observational work on the wards, supplemented by qualitative interviews and many emails led us to question the relationship between this ‘consumption’ data and overall consumption patterns. We know, for example, that since laboratory results have been available, many women are getting higher-end antibiotics and that this is contributing to shortened stays and improved patient outcomes [10]. However, the supplies of these drugs were not visible in the data. It seems that the pharmacists have played a critical role in supporting access to these antibiotics through a combination of ‘borrowing’ from other hospital supplies. The following excerpt explains this process:

There is a TB focal person who handles all TB related logistics, including the ordering of TB drugs. These drugs are stored in main stores. Rx only focuses on drugs from NMS. In most cases, we don’t have the changed antibiotics in stock or in our procurement plan or we have limited quantities. Take Amikacin for example. We usually borrow from the TB program and give to the patients. The same applies to Moxifloxacin. These medicines are not available in the inpatient pharmacy. In fact, one time you had to use K4C money to purchase Amikacin. Because at times we have septic patients who are only responsive to these drugs, we ‘beg’/borrow from the TB drugs. Apparently, this has caused audit queries.

This ‘borrowing’ behaviour clearly saves women’s lives; it also compromises the pharmacists under pressure to assist, but potentially contravening donor conditionalities. We can anticipate similar situations in relation to anti-retroviral therapies (for HIV patients) and also, in the case of FPRRH given its proximity to Congo, some (necessary) stockpiling in the event of Ebola spread.

Other apparent ‘discrepancies’ in the data, including very sporadic and low use of antibiotics in the neo-natal intensive care unit (NICU) and the paediatric ward uncovered other variances in practice, which are undocumented and apparently do not comply with the published protocol. This was explained by one respondent as follows:

The paediatric ward gets injectable drugs (directly) from main stores, including antibiotics, but oral antibiotics and other oral drugs from in-patient pharmacy.

When drugs are prescribed but not in-stock, patients are asked to buy them privately. Current recording systems cannot capture the consumption of privately purchased drugs. These examples underline the need to exercise caution when interpreting data on antibiotic consumption. The team is currently completing a more in-depth SSI follow-up on patients which will capture some of these processes.

## 4. Summary and Conclusions

Dyar’s paper [24] reviews the use of the term ‘antimicrobial stewardship’ and concludes that there has been an overemphasis on conceptualisations focused on ‘individual prescriptions’, and insufficient emphasis on the societal implications of antimicrobial use. Furthermore, and of particular relevance to the MSI project, there has been insufficient translation of the concepts of ‘responsible use’ into context and time-specific actions. The authors conclude that AMS is not so much a concept, as it is a tool to assess whether organisations are identifying actions to improve responsible use in the specific context within which they are functioning. This idea fits very well with the action-research approach used in our intervention, and the results arising from that.

Sadly, the changes described above are essential to achieving ‘responsible use’ in a Ugandan RRH; but they are not sufficient. They create the opportunity for active pharmacy engagement in multi-disciplinary decision-making. This achievement could have a major impact on AMS at the hospital, as these wards consume by far the largest volume of antibiotics at the hospital. The cost-effectiveness of this intervention underlines the sustainability potential and the immediate opportunity for scale-up across the hospital as a whole, but also to other public health facilities in Uganda and beyond. Cox et al. make the important point that, ‘delayed or no access to antibiotics kills more people than antibiotic resistant bacteria. … AMS is not only about reducing inappropriate use, but also assuring access to effective treatment’ [1] (p. 813).

The findings evidence significant and impactful behaviour change on the PNG wards, with genuine multi-disciplinary team-working contributing to changes in prescribing behaviour and AMS. Wound care and laboratory testing lie at the heart of these changes centre staging nurses, midwives and laboratory scientists in AMS processes. The results of the microbiology testing then provide a platform for genuine multi-disciplinarity and, specifically, the first opportunity for clinical pharmacy engagement. This is true both at the level of rational (evidence-based) prescribing for individual patients and in improving the evidence base behind empirical prescribing (through an understanding on patterns of AMR). If access to a full range of antibiotics were available, this platform of behaviour change would transform antimicrobial use patterns, reduce the overuse and inappropriate use of antimicrobials and improve patient outcomes and deliver significant cost-benefits.

The second part of the paper has elaborated the complexity and opacity of the supply chain system in a RRH setting in Uganda. In the absence of effective supplies not only will these cost-savings elude facilities, but the patients involved will fail to thrive, and the motivation of health workers to apply the skills and knowledge they have demonstrated will inevitably decline. The paper has explained, in detail, the complex dynamics of supply chain management in a Ugandan public hospital. Understanding and piecing together these processes has required painstaking ethnographic research to unpick major errors in record-keeping and interpret the trends observed. In the first instance, the very centralised system creates huge dependency on the functionality of NMS and the adequacy of Ministry budgets. Centralisation may be seen as necessary in systems so damaged by corruption but where this undermines flexibility and responsiveness and generates extended and predictable stock-outs, the systems put in place to improve AMS will, inevitably, fail. 

The MSI has demonstrated the potential for change and the efficiencies associated with this. We hope that publication of this evidence will stimulate discussion at national level amongst all key stakeholders, and generate a momentum for change. The COVID-19 pandemic has thrown a light on supply-chain effectiveness and the impact of weak supply chains on global and national inequalities. Although the poorest in societies will suffer disproportionately, the tentacles of AMR, as with all global pandemics will reverberate across the globe.

At a local level, after years of ongoing engagement, the Kabarole Health Partnership has recently signed a Public-Private-Partnership (PPP) agreement, which was triggered by the current project. The PPP will generate a more sustainable and integrated mechanism for supply-chain augmentation with an emphasis on IPC and antimicrobials. This will enable foreign organisations to cooperate on a co-decision and co-funding basis, guided by the hospital’s Medicine Therapeutic Committee, and supported by a not for profit supplier, Joint Medical Stores. The objective will be to move away from dependency-generating donations to a more integrated approach with the agility to respond to local needs.

## Figures and Tables

**Table 1:** Volume and Proportion of Suspected Sepsis Cases Sent for Laboratory Testing.

Time Frame	Suspected Sepsis Cases	Culture and Sensitivity Tests Performed	% Tested
1st January 2019–8th July 2019	50	0	0%
9th July 2019–21st July 2019	16	3	19%
22nd July 2019–31st January 2020	76	74 (2 had missing data)	95%

**Table 2:** The Order Fill Rate at Fort Portal Regional Referral Hospital (FPRRH) (2019/2020).

Financial Year Cycle	Total Items Ordered	Total Items Delivered	Fill Rate
CYCLE 1 (July–August 2019)	307	236	77%
CYCLE 2 (September–October 2019)	306	232	76%
CYCLE 3 (November–December 2019)	309	226	73%

Source: Rx on-line medicines management system (National Medical Stores (NMS) do not provide data on fill rates for specific medications)

## Supplementary Materials

The following are available online at https://www.mdpi.com/2079-6382/9/6/315/s1, File 1: Antimicrobial Resistance patterns at FPRRH. Figure 1: Extract from the 2020/2021 Procurement Plan (with bi-monthly figures) focusing on key Antibiotics used on Post Natal and Gynae Wards, Figure 2: Supply of Oral Antibiotics to All Wards in January and February 2020, Figure 3: Dispensing of IV Ceftriaxone and Metronidazole from In-Patient Pharmacy to PNG Wards (1/12/2019–29/03/2020), Figure 4: Dispensing of Oral Amoxicillin and Metronidazole from In-Patient Pharmacy to PNG Wards (1/12/2019–29/03/2020).
